# Reduction of Huntington’s Disease RNA Foci by CAG Repeat-Targeting Reagents

**DOI:** 10.3389/fncel.2017.00082

**Published:** 2017-03-28

**Authors:** Martyna O. Urbanek, Agnieszka Fiszer, Wlodzimierz J. Krzyzosiak

**Affiliations:** Department of Molecular Biomedicine, Institute of Bioorganic Chemistry Polish Academy of SciencesPoznan, Poland

**Keywords:** Huntington’s disease, polyglutamine diseases, RNA foci, RNA toxicity, siRNA, antisense oligonucleotides

## Abstract

In several human polyglutamine diseases caused by expansions of CAG repeats in the coding sequence of single genes, mutant transcripts are detained in nuclear RNA foci. In polyglutamine disorders, unlike other repeat-associated diseases, both RNA and proteins exert pathogenic effects; therefore, decreases of both RNA and protein toxicity need to be addressed in proposed treatments. A variety of oligonucleotide-based therapeutic approaches have been developed for polyglutamine diseases, but concomitant assays for RNA foci reduction are lacking. Here, we show that various types of oligonucleotide-based reagents affect RNA foci number in Huntington’s disease cells. We analyzed the effects of reagents targeting either CAG repeat tracts or specific *HTT* sequences in fibroblasts derived from patients. We tested reagents that either acted as translation blockers or triggered mRNA degradation via the RNA interference pathway or RNase H activation. We also analyzed the effect of chemical modifications of CAG repeat-targeting siRNAs on their efficiency in the foci decline. Our results suggest that the decrease of RNA foci number may be considered as a readout of treatment outcomes for oligonucleotide reagents.

## Introduction

A group of human neurodegenerative diseases is caused by the expansion of CAG repeats located in the coding sequence of single functionally unrelated genes; these disorders are called polyQ diseases. The most thoroughly analyzed member of this group of disorders is HD, caused by expanded CAG repeats in the first exon of the *HTT* gene. Both RNA and protein products from the mutant allele are proposed to be involved in the pathogenic process; therefore, the most promising therapeutic approaches are designed to target the mutant transcripts. Mutant mRNA is partially or temporarily retained in the nucleus within splicing speckles, as shown in multiple types of cellular models of polyQ diseases (De Mezer et al., 2011; Urbanek et al., 2016). Increased retention of mutant RNA in the nucleus is associated with compromised functions of proteins that bind to mutant RNA (Jazurek et al., 2016); examples of such malfunctions are alternative splicing abnormalities (Mykowska et al., 2011; Sathasivam et al., 2013; Cabrera and Lucas, 2016) and other gene expression alterations (Sharma and Taliyan, 2015). For that reason, RNA foci are increasingly considered undesired toxic structures, rather than a protective cellular self-defense mechanism. RNA foci were shown in various types of cellular models of HD, including fibroblasts, lymphoblasts, and neuronal progenitors (Urbanek and Krzyzosiak, 2016).

Various approaches have been used to decrease RNA foci in different repeat expansion diseases, including myotonic dystrophy type 1 (DM1), myotonic dystrophy type 2, fragile X–associated tremor/ataxia syndrome, and amyotrophic lateral sclerosis and frontotemporal dementia (ALS-FTD) (Supplementary Table S1) (Langlois et al., 2003; Disney et al., 2012; Nguyen et al., 2014; Rzuczek et al., 2014; Su et al., 2014). The most widely analyzed foci-targeting factors are small molecules that decrease the nuclear accumulation of mutant RNA and significantly reduce pathological interactions of mutant RNAs with proteins in the case of DM1 (Warf et al., 2009; Jahromi et al., 2013a,b; Hoskins et al., 2014; Ketley et al., 2014; Wojciechowska et al., 2014; Wong et al., 2014). However, various ON reagents have also been used successfully for DM1 and ALS-FTD (Mulders et al., 2009; Wheeler et al., 2009, 2012; François et al., 2011; Larsen et al., 2011; Lee et al., 2012; Lagier-Tourenne et al., 2013; Sareen et al., 2013; Sobczak et al., 2013; Wojtkowiak-Szlachcic et al., 2015). Multiple therapeutic approaches have been proposed for polyQ diseases (reviewed in Fiszer and Krzyzosiak, 2014; Keiser et al., 2016), but their effects on nuclear RNA foci have been barely analyzed. To date, only a single recent report refers to foci reduction, showing that 20-nt-long LNA-modified ON composed of CTG units disrupts CAG RNA foci in an HD model (Rué et al., 2016).

The ON-based reagents tested to develop therapeutic approaches for polyQ diseases include mostly siRNAs and ASOs. siRNAs localize mainly in the cytoplasm, which is their primary site of action. However, siRNAs can also function in the nucleus (Robb et al., 2005; Gagnon et al., 2014), reviewed in Kalantari et al. (2016). In contrast, ASO reagents are thought to function mainly in the nucleus, activating RNase H, although, they can also be active in the cytoplasm (Castanotto et al., 2015; Liang et al., 2015). The localization of reagents may determine their cellular functionality, especially when target transcripts are captured in distinct structures.

An attractive therapeutic approach is targeting the mutation site directly in transcripts implicated in polyQ diseases. In order to achieve high preference in the silencing of mutant alleles, CAG repeat-targeting siRNAs have been modified to form base mismatches with the target sequence and induce a mechanism similar to that of miRNAs (Hu et al., 2010; Fiszer et al., 2011, 2013, 2016; Yu et al., 2012; Liu et al., 2013). In mutation-targeting approach also oligomers acting as translational blockers were developed as potential therapeutics for HD and spinocerebellar ataxia type 3 (SCA3) (Hu et al., 2009; Fiszer et al., 2012). For targeting specific sequence of huntingtin mRNA, siRNAs, and ASOs were successfully tested in HD mouse models (Wang et al., 2005; DiFiglia et al., 2007; Boudreau et al., 2009; Carroll et al., 2011; Kordasiewicz et al., 2012; Ostergaard et al., 2013; Southwell et al., 2014).

In this study, we analyzed the influence of ON-based reagents on RNA foci observed in HD fibroblasts. We aimed to establish whether activity pathways of ONs affect their potential to decrease RNA foci and whether ASO ONs, RNAi triggers, or LNA blocker are more effective in decreasing nuclear foci. We also aimed to compare the activity of different chemically modified ONs and to examine the correlation between their inhibitory activity on RNA and protein expression and their potential to decrease RNA foci number. To gain deeper insight into the mechanism of ON action we considered following functionality scenarios:(I) non-functional reagents that neither affect the size, number, and morphology of foci nor protein or RNA levels; (II) reagents that decrease RNA and protein levels, but do not affect RNA foci, indicating the degradation of RNA only in the cytoplasm; (III) reagents that decrease only protein levels, acting as translation blockers; (IV) reagents that decrease RNA foci as well as protein and RNA levels, indicating the degradation of transcripts in both the nucleus and cytoplasm; (V) reagents that decrease the foci number, but increase RNA and protein levels, suggesting the release of transcripts from foci to the cytoplasm, without further degradation; and (VI) reagents that increase the number of RNA foci by triggering the retention of RNA in the cell nucleus.

## Materials and Methods

### Cell Culture

Human fibroblasts (control: GM07492; HD: GM04281, 17/68 CAG in *HTT* gene, HD2: GM09197, 21/151 CAG in *HTT* gene) were obtained from Coriell Repository (Camden, NJ, USA). Fibroblasts were cultured in Minimal Essential Medium (Sigma–Aldrich, St. Louis, MO, USA) with 10% Fetal Bovine Serum (Sigma–Aldrich) supplemented with GlutaMAX (Life Technologies, Carlsbad, CA, USA), non-essential amino acids (Sigma–Aldrich), and Antibiotic Antimycotic Solution (Sigma–Aldrich) at 37°C. All cell cultures were checked for mycoplasma contamination.

### Oligonucleotides and Cell Transfection

Oligonucleotide were synthesized by Future Synthesis (Poznan, Poland) or IDT (Coralville, IA, USA), and duplexes were annealed according to the manufacturer’s instructions. The LNA oligomer was synthetized by Exiqon (Vedbæk, Denmark). The sequences of the synthetic ONs and oligomer used in this study are presented in **Table 1**.

**Table 1:** The nucleotide sequences and chemical modifications of the ONs tested in this study.

Oligonucleotide (5′–3′)	Main mechanism of action	Comment/Reference
**CAG repeat tract-specific**		
**A2** AS: GCUGCUGC**A**GCUGCUGCUGCU	RNAi/miRNA	Fiszer et al., 2013
**G2** AS: GCUGCUGC**G**GCUGCUGCUGCU	RNAi/miRNA	Fiszer et al., 2013
**A2F** AS: 5′P GCUGCUGC**A**GCUGCUGCUGCU	RNAi/miRNA	Fiszer et al., 2016
**A2M** AS: 5′P G^∗^C^∗^UG^∗^CU^∗^GC^∗^**A**G^∗^CU^∗^GC^∗^U^∗^G^∗^C^∗^U^∗^G^∗^C^∗^U	RNAi/miRNA	Modifications pattern corresponding to Yu et al., 2012/Fiszer et al., 2016
**CAG/CUG** AS: CUGCUGCUGCUGCUGCUGCUGC SS: CAGCAGCAGCAGCAGCAGCAGC	RNAi	
**LNA CTG** (C)(T)(G)(C)(T)(G)(C)(T)(G)(C)	Blocker	Corresponding to CAG LNA tested by Wojtkowiak-Szlachcic et al., 2015
**ASO CTG** C^∗^U^∗^G^∗^C^∗^U^∗^g^∗^c^∗^t^∗^g^∗^c^∗^t^∗^g^∗^c^∗^t^∗^g^∗^C^∗^U^∗^G^∗^C^∗^U	RNaseH	
**HTT sequence-specific**		
**siHTT** AS: ACUUGAGGGACUCGAAGGCCU SS: GGCUUCGAGUCCCUCAAGUCC	RNAi	Fiszer et al., 2013
**ASO HTT** U^∗^C^∗^U^∗^C^∗^U^∗^a^∗^t^∗^t^∗^g^∗^c^∗^a^∗^c^∗^a^∗^t^∗^t^∗^C^∗^C^∗^A^∗^A^∗^G	RNaseH	Carroll et al., 2011
**Other**		
**siLuc** AS: UCGAAGUAUUCCGCGUACGUU SS: CGUACGCGGAAUACUUCGAUU	RNAi	siRNA with no target in cells
**BLOCK-iT Fluorescent Oligo** (Life Technologies)	Control	Transfection efficiency control


*Base substitutions in CAG repeat tract-specific ONs, resulting in mismatch formation with the target sequence, are marked in bold. Legend for the composition and chemical modifications of ONs: N – RNA; n – DNA; 5′P – 5′-phosphorylation; N – 2′OMe; ^∗^ – PTO; N – 2′F; (N) – LNA.*

Cell transfections were performed using Lipofectamine 2000 (Life Technologies) according to the manufacturer’s instructions. The reagents were transfected at 50 nM, unless stated otherwise in figure legends. Oligonucleotide treatment lasted for 4 h; after that time medium was changed. Material for subsequent analyses was collected after 48 h. For fixation, cells were seeded directly on cover slips prior to transfection. The transfection efficiency was monitored using BlockIT fluorescent siRNA (Life Technologies).

### RNA Fluorescence *In situ* Hybridization and Immunofluorescence

Fluorescence *in situ* hybridization and IF procedures were performed as described previously (Urbanek and Krzyzosiak, 2016). Briefly, cells were fixed in 4% paraformaldehyde at 4°C for 30 min. Permeabilization was performed with 2% acetone for 5 min, and overnight incubation in 70% EtOH. Prior to hybridization, cells were prehybridized in 30% formamide, 2x SSC buffer for 30 min at RT. Hybridization was performed in hybridization buffer (30% formamide, 2x SSC, 200 ng/ml ssDNA, 0.02% BSA, 10% dextran sulfate, 2 mM vanadyl-ribonucleoside, 2 nM probe) at 37°C overnight. Washing was performed with 30% formamide, 2x SSC and 1x SSC at RT. For biotin-labeled probes washings were performed only in 1x SSC and subsequently samples were blocked with 1% BSA buffer for 1 h at RT and incubated with IgG Fraction Monoclonal Mouse Anti-Biotin antibody (1:200, 200-542-211, Jackson ImmunoResearch; West Grove, PA, USA) overnight at 4°C. For signal enhancement samples were additionally incubated for 1 h with AffiniPure F(ab′)_2_ Fragment Donkey Anti-Mouse IgG (H+L) antibody labeled with Alexa488 (1:500, 715-546-150, Jackson ImmunoResearch; West Grove, PA, USA). For RNase treatment, cells were subjected to 0.01 μg/ml RNase A for 1.5 h after the permeabilization step. For IF, samples were blocked with 1% BSA buffer for 1 h at RT, incubated with primary anti-huntingtin antibody (1:200, MAB2166, Millipore) at 4°C overnight and with secondary antibody AffiniPure F(ab′)_2_ Fragment Donkey Anti-Mouse IgG (H+L) antibody labeled with Alexa488 (1:500) for 1 h. For combined RNA FISH and IF, first, FISH was performed with only 1x SSC washings. Next, samples were blocked with 1% BSA for 30 min and incubated with anti-huntingtin antibody for 3 h and secondary antibody for 45 min. For endosomes visualization anti-Rab5 (1:200, 12666T, Cell Signaling Technology; Danvers, MA, USA) and anti-EEA1 (1:100, 12666T, Cell Signaling Technology) antibodies and secondary antibody AffiniPure F(ab′)_2_ Fragment Donkey Anti-Rabbit IgG (H+L) antibody labeled with Alexa488 (1:500, 711-546-152, Jackson ImmunoResearch) were used. Nuclei were imaged with SlowFade Gold with DAPI (Life Technologies). Images were captured with a Leica SP5 confocal microscope.

For CAG-specific ONs detection in cells we used DNA probe Cy3-(CAG)_6_CA (Metabion, Germany), for foci imaging we used either LNA-modified DNA probe Cy3-(CTG)_6_CT (Exiqon, Denmark) or three *HTT*-specific 5′-Biotin-labeled DNA probes: GGTAAAAGCAGAACCTGAGCGGCCGTCCATCTTGGACCCGT, TCGAAGGCCTTCATCAGCTTTTCCAGGGTCGCCAT, TCCATAGCGATGCCCAGAAGTTTCTGAAATTCTGGAG (Metabion).

### Western Blot Analysis

The western blot analysis for the HTT protein (17/68Q tract) was performed as previously described (Fiszer et al., 2011). Briefly, 30 μg of total protein was run on a Tris-acetate sodium dodecyl sulfate (SDS)-polyacrylamide gel (1.5 cm, 4% stacking gel/4.5 cm, 5% resolving gel, acrylamide:bis-acrylamide ratio of 49:1) in XT Tricine buffer (Bio-Rad, Hercules, CA, USA) at 130 V in an ice-water bath. Subsequently, the proteins were wet-transferred to a nitrocellulose membrane (Sigma–Aldrich). All of the immunodetection steps were performed using the SNAPid system (Millipore). The primary antibodies anti-huntingtin (1:1000, MAB2166, Millipore) and anti-plectin (1:1000, ab83497, Abcam, Cambridge, UK) and secondary antibodies anti-mouse HRP-conjugate (1:2000, A9917, Sigma–Aldrich) and anti-rabbit HRP-conjugate (1:2000, 711-035-152, Jackson ImmunoResearch) were used in a PBS/0.1% Tween-20 buffer containing 0.25% non-fat milk. The immunoreaction was detected using WesternBright Quantum HRP Substrate (Advansta, Menlo Park, CA, USA). The protein bands were scanned directly from the membrane using a camera and were quantified using Gel-Pro Analyzer.

### RNA Isolation and Reverse Transcription-Polymerase Chain Reaction

Total RNA was isolated from fibroblast cells using TRIzol reagent (Sigma–Aldrich) and a Direct-zol Kit (Zymo Research, Irvine, CA, USA) according to the manufacturer’s instructions. For isolation of RNA fractions Cytoplasmic and Nuclear RNA Purification kit (Norgen Biotek, Corp., Thorold, ON, Canada) was used according to the manufacturer’s instructions. The RNA concentration was measured using a DeNovix spectrophotometer (Wilmington, DE, USA). An amount of 500 ng of total RNA or 200 ng of fractionated RNA was reverse transcribed at 55°C using Superscript III (Life Technologies) and random hexamer primers (Promega, Madison, WI, USA). cDNA was used for qPCR using LightCycler 480 SYBR Green I Master (Roche, Basel, Switzerland) with denaturation at 95°C for 10 min, followed by 45 cycles of denaturation at 95°C for 10 s, annealing at 60°C for 15 s, and elongation at 72°C for 20 s, with *HTT*, *GAPDH*, or *U6*-specific primers (sequences are listed in Supplementary Table S2) on the Light Cycler 480 II (Roche). Data pre-processing and normalization were performed using LightCycler 480 SW 1.5.1 software.

### Image Analysis

ImageJ software (Fiji distribution) was used for image analysis. Images were adjusted with respect to brightness, contrast, and smooth effects. For the statistical analysis of images, a Python script for ImageJ was prepared.

#### Number of Foci Estimation

For estimation of RNA foci number Python (Jython) script was prepared for ImageJ. First, analyzed area was restricted to the nucleus using DAPI signal. Next, after adjusting threshold for the red signal (from probe), image was converted to mask and particles were analyzed (with restrictions to minimal size of foci but accepting all shapes with circularity index). Each image represented single cell. Results for group of images were saved in text file for statistical analyses.

#### Quantity of Nuclear Transcripts

For estimation of quantity of nuclear CAG transcripts Python (Jython) script was prepared for ImageJ. First, analyzed area was restricted to the nucleus using DAPI signal. Next, mean intensity signal (red canal from the probe) from the nucleus was calculated. Each image represented single cell. Results for group of images were saved in text file for statistical analyses.

### Statistical Analysis

All experiments were repeated at least three times. The statistical significance of changes in gene expression levels (real-time PCR, western blotting, and IF level) was assessed using a one-sample *t*-test, with an arbitrary value of 1 assigned to cells treated with control siRNA (siLUC). Selected data were compared using an unpaired *t*-test with Welch’s correction to assess the allele-selectivity of silencing (normal vs. mutant allele silencing). The statistical significance of the number of foci, nuclear RNA signal and percent of aggregate-positive cells was assessed using a one-way ANOVA and multiple comparisons testing with *post hoc* Dunnett’s tests. For FISH and IF analyses, at least 30 cells were analyzed for each experimental condition. *p*-values of <0.05 were considered significant.

## Results

### Selection and Design of Oligonucleotides

We used a set of chemically synthesized ONs that differed with regard to their (I) targeted sequence (specific *HTT* sequence or CAG repeats), (II) anticipated mechanism of activity (RNAi-based, RNase H-inducing, and a “blocker”), and (III) pattern of chemical modification (pure RNA ONs and RNAs containing 2′-fluoro (2′F), 2′-*O*-methylo (2′OMe), and phosphorothioate (PTO) modifications, DNA gapmers with 2′OMe and PTO, as well as LNA oligomer). All ON sequences are presented in **Table 1** with references if they were used in previous studies. Specifically, we used siRNA and ASO targeting *HTT*-specific sequences (siHTT and ASO HTT, respectively) and a set of CAG repeat-targeting ONs: unmodified RNA duplex (CAG/CUG), miRNA-like siRNAs with single mismatches (A2 and G2), chemically modified siRNA (A2F and A2M), antisense oligonucleotide (ASO CTG) and a short LNA blocker (LNA CTG) (**Figure 2A**).

### CAG-Targeting RNA Interference Reagents Localize Predominantly to the Cytoplasm and Antisense ONs to the Nucleus

**FIGURE 1:**
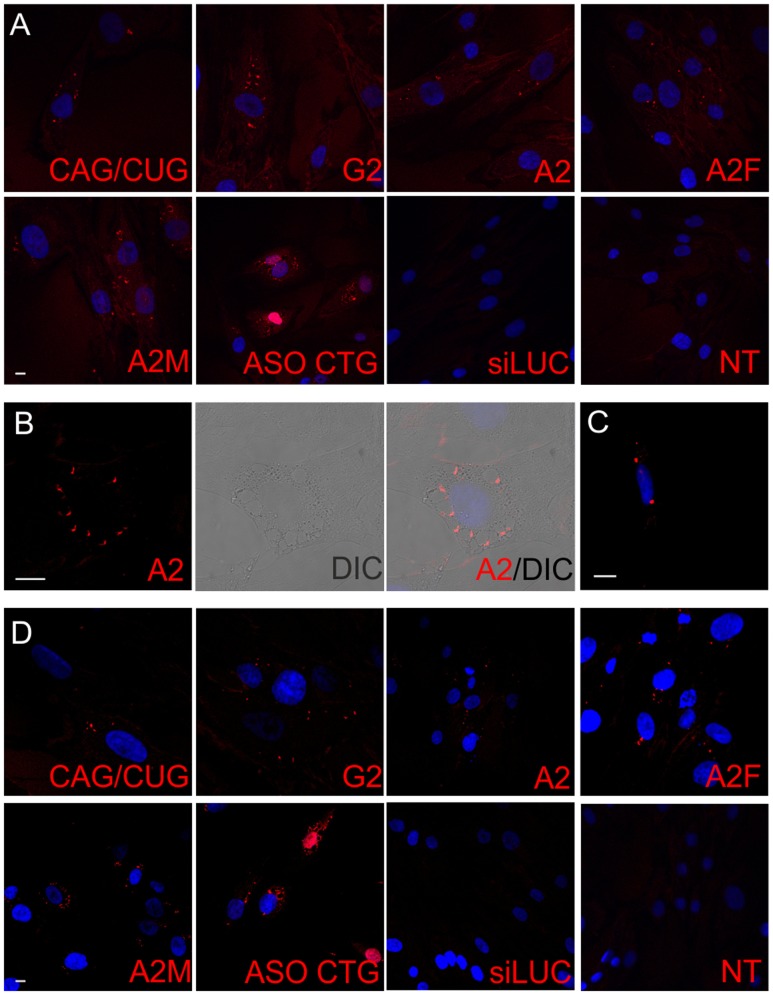
**Localization of siRNAs and ASOs targeting CAG repeat tract in fibroblasts.** **(A)** Representative RNA FISH images of the control fibroblast cell line after transfection with indicated ONs are presented. **(B)** Localization of A2 reagent in control fibroblasts. **(C)** Localization of the passenger strand from CAG/CUG duplex in control fibroblasts. **(D)** Representative RNA FISH images of the HD fibroblast cell line after transfection with indicated ONs. DAPI staining (blue), reagents (red); bar = 10 μm.

First, we aimed to determine the cellular distribution of ONs targeting CAG repeat sequences. Selected siRNAs (CAG/CUG, A2, G2, A2F, and A2M), ASO CTG, and LNA CTG were delivered by lipid-based transfection. After 48 h, cells were fixed and small-RNA FISH was performed using probes that interact with the ONs in a 1:1 stoichiometry. However, we were not able to visualize LNA reagents owing to their short length. First, we obtained images of reagents in control fibroblasts (**Figure 1A**). We observed predominantly cytoplasmic localization of RNA interference reagents, both unmodified and chemically modified. Owing to the differences in binding strengths of the probes to various ONs, we did not quantify differences between reagents. The ONs mainly localized to cytoplasmic vesicles (**Figure 1B**), similar to the localization observed for control fluorescent siRNA, BlockIT (data not shown). Observed ONs did not localize within endosomes marked with EEA1 and Rab5 proteins showing that these are probably vesicles formed by the lipofection (Supplementary Figures S1A,B). It is worth noting that using this experimental approach, we could observe only cellular spots that contained multiple ON molecules, but not the localization of single molecules that could represent sites of interactions with target sequences. We also observed the passenger strand of CAG/CUG, which showed similar localization to that of the guide strand (**Figure 1C**). ASO localized within both the cytoplasm and nucleoplasm; however, a strong bias toward the nucleus was observed. Non-transfected cells and cells transfected with control siRNA siLUC were used as negative controls. Next, we examined whether the CAG repeat-expanded tract present in HD cells affects the localization of reagents targeting the CAG sequence. We did not observe significant differences in localization between HD and control fibroblasts, indicating that the presence of the mutation in cells does not alter reagent localization (**Figure 1D**).

### Additional Assays to Estimate Cellular Effects of ON Treatments

**FIGURE 2:**
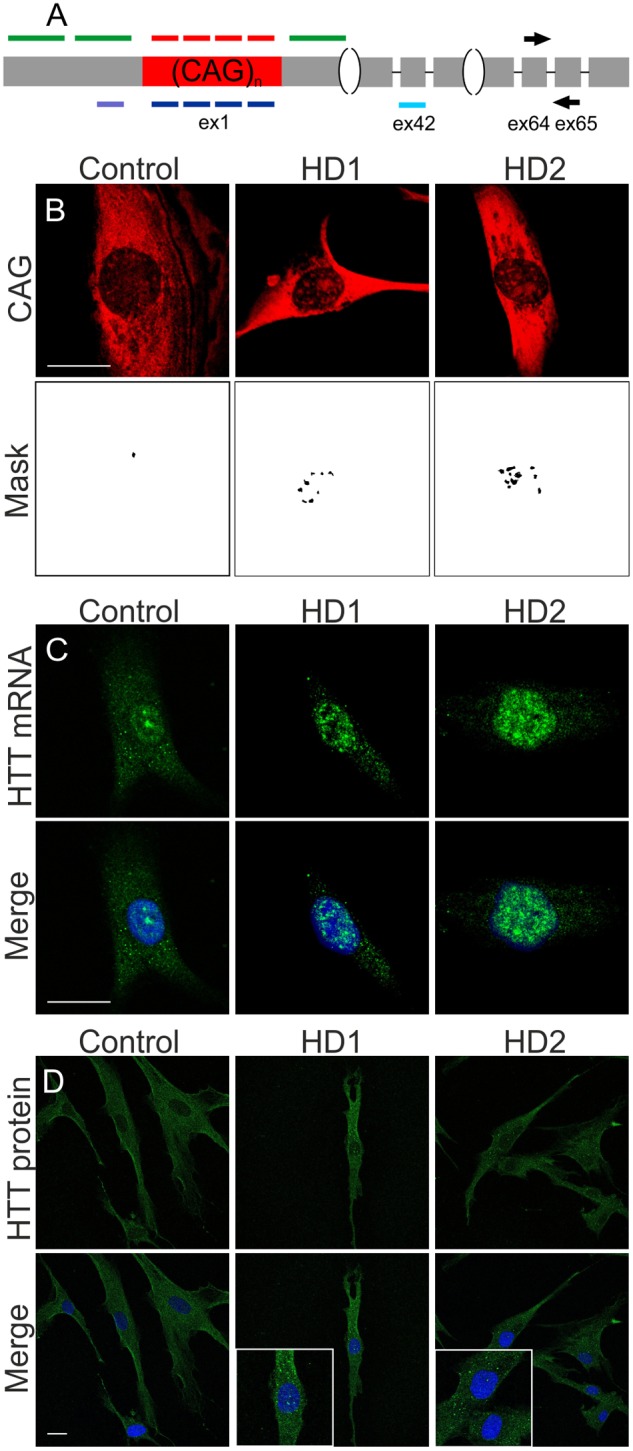
**Imaging systems developed for monitoring effects triggered by ONs in cells.** **(A)** Scheme of *HTT* mRNA with place of probes binding (red: CTG probe, green: *HTT*-specific probes) and ONs binding (navy: CAG-targeting ONs, violet: siHTT, turquoise: ASO HTT). Scheme shows also the binding sites of the primers used for PCR amplification on the junction of *HTT* exon 64 and 65. Gray boxes represent exonic sequence and the black lines represents introns. **(B)** RNA foci visualized with CAG-specific probe in HD cells with mask used to count RNA foci, bar = 20 μm. **(C)** RNA foci visualized with *HTT*-specific probes in HD cells, bar = 20 μm. **(D)** Huntingtin-specific IF to visualize protein levels and aggregates in HD cells, bar = 25 μm. Representative images of control, HD (17/68 CAG), and HD2 (21/151 CAG) fibroblast cells are presented. DAPI staining (blue), *HTT* transcripts (green), HTT protein (green), CAG repeats (red).

In our earlier studies we defined the effectiveness of ON treatment at the RNA level using RT-PCR (separate analyses for normal and mutant alleles) and at the protein level using western blot (Fiszer et al., 2011, 2013, 2016). To monitor treatment outcomes more broadly, we added in the current study a microscopic analyses of CAG RNA foci and huntingtin levels and localization. We used a CTG probe that was demonstrated in previous studies (Urbanek and Krzyzosiak, 2016) to successfully visualize RNA foci in cellular models of polyQ diseases (**Figure 2B**). Both analyzed HD lines demonstrated nuclear RNA foci, which were observed in about 50% cells. RNA foci were not detected after RNase treatment (Supplementary Figure S2). As shown for LNA reagents, for which their binding to repeat sequences may block subsequent RT-PCR (Rué et al., 2016), we demonstrated that the reagents did not interfere with the RNA FISH procedure by visualizing *HTT* mRNA with *HTT* sequence-specific probes. Probes targeting the first exon of *HTT* mRNA were used to successfully visualize RNA foci in HD fibroblasts. In control fibroblasts, the signal was rather uniform within the nucleoplasm and cytoplasm, with increased signals strength observed in nucleoli (**Figure 2C**). An additional feature of HD that may serve as an indicator of an effective therapeutic approach is the presence of HTT protein aggregates. We observed huntingtin aggregates in the cytoplasm and nucleus in both HD fibroblast cell lines, and no such aggregates were visible in the control cell line (**Figure 2D**). Thus in addition to western blotting, IF may be used to observe protein level changes resulting from ON treatment.

### Effects of ON-Based Reagents on HTT Expression at the mRNA and Protein Levels

**FIGURE 3:**
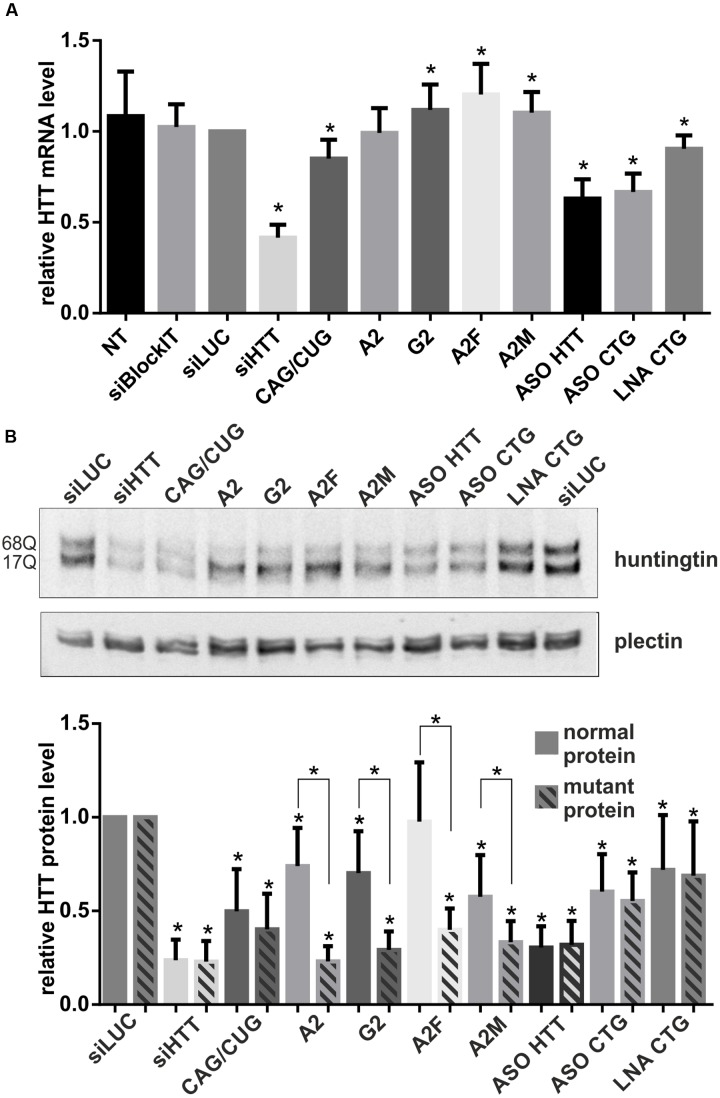
**Regulation of *HTT* expression by ONs.** **(A)** qRT-PCR analysis of total *HTT* mRNA levels in HD fibroblasts after transfection with 50 nM of the indicated ONs. *HTT* level was normalized to the *GAPDH* level. **(B)** Western blot analysis of normal and mutant huntingtin levels for the same experiment as in **(A)**. The level of HTT alleles was normalized to plectin level. In all samples expression level was referred to *HTT* expression in cells transfected with siLUC (set as 1). NT – non-treated cells. The *p-*value is indicated with an asterisk (^∗^*p* < 0.05); graphs are presented with standard deviation values.

We investigated the silencing of *HTT* expression at the mRNA and protein levels using selected ONs under the same experimental conditions as used for microscopic analyses, i.e., 48 h after the transfection of HD fibroblasts with 50 nM ONs. We assessed total *HTT* mRNA levels using qRT-PCR and primers located downstream the CAG repeat tract (**Figures 2A**, **3A**). The most significant decreases in *HTT* mRNA were observed for siHTT (to ∼40% of the control level) and for both ASOs (to ∼65% of the control level). CAG/CUG reagent decreased level of *HTT* mRNA to ∼85%. A separate analysis of *HTT* alleles by semi-quantitative RT-PCR revealed a similar general trend of *HTT* silencing by these ONs and there was no indication of allele-selective inhibition by ASO reagents (data not shown). At the selected time point, we did not observe decrease in *HTT* mRNA level by A2, G2, A2F, and A2M ONs. Additionally, for selected set of ONs we performed RNA fractionation to analyze changes in nuclear and cytoplasmic transcript level (Supplementary Figures S3A,B). We tested siHTT, A2, ASO HTT, and ASO CTG in this assay. The results were generally consistent with qRT-PCR for total transcript level described above. siHTT caused lowering of *HTT* mRNA already in the nucleus (to ∼70%) but more prominent effects were observed in the cytoplasm what in total resulted in *HTT* mRNA decrease to ∼25%. For A2 we observed slight increase of *HTT* transcript in the nucleus and slight decrease in the cytoplasm. As expected, the effects of ASOs activity were mainly nuclear as these ONs decreased *HTT* mRNA level to ∼45% already in the nucleus. For all nine ONs we performed western blotting to analyze normal and mutant huntingtin levels separately (**Figure 3B**). For ONs that lowered *HTT* mRNA (siHTT and ASOs), significant decreases in protein levels were also observed, to ∼25%, ∼30%, and ∼60% of the control level for siHTT, ASO HTT and ASO CTG, respectively. Neither siHTT, nor any of the used ASOs acted in an allele-selective way, decreasing both alleles with similar strength. Consistent with previous reports, selected CAG repeat-targeting reagents caused the allele-selective lowering of huntingtin, with a high preference for the mutant protein observed for A2, G2, and A2F. Significant allele-selectivity was also observed for A2M ON, but not for CAG/CUG siRNA or LNA CTG.

### Effects of ON-Based Reagents on RNA Foci

**FIGURE 4:**
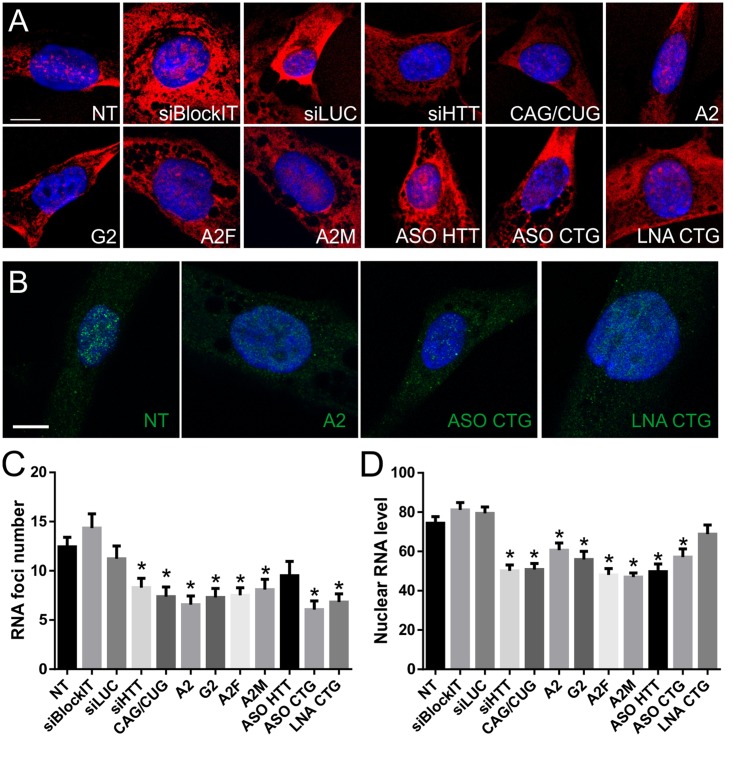
**Nuclear RNA level and foci number reduction after ON treatment of HD fibroblasts.** **(A)** Representative RNA FISH images of HD fibroblast cells treated with indicated ONs visualized with CAG-specific probe. **(B)** Representative RNA FISH images of HD fibroblast cells treated with indicated ONs visualized with *HTT*-specific probes. DAPI staining (blue), CAG repeats (red), *HTT* mRNA (green); bar = 10 μm. **(C)** Number of RNA foci per cell, calculated from images obtained using CAG-specific probe (see Number of Foci Estimation). **(D)** Nuclear level of transcript (represented as mean signal intensity per cell), calculated from RNA FISH images based on nuclear signal from CAG-specific probe (see Quantity of Nuclear Transcripts). Statistical significance of changes observed after ON treatment was assessed in reference to measurements obtained for NT (non-treated) cells. The *p*-value is indicated with an asterisk (^∗^*p* < 0.05); graphs are presented with standard error of mean (SEM) values.

Next, we analyzed RNA foci after ONs treatment to investigate whether mRNAs within the nucleus are accessible to ON reagents, whether these ONs exert their activity within the nucleus, and whether they can decrease RNA foci. Our results showed that not all tested ONs are able to significantly decrease the number of RNA foci, despite altering the RNA or protein levels. The results differed for probes used; however, a similar tendency to reduce number of RNA foci was observed (**Figures 4A,B**). Untreated HD fibroblasts had a mean of 12.4 foci per foci-positive cell, in agreement with our previous reports. Cells treated with siLUC and BlockIT exhibited similar means, with 11.2 and 14.3 foci per cell, respectively. Most significantly RNA foci-disrupting ONs were A2, with a mean of 6.6 foci per cell, ASO CTG, with a mean of 6.1, and LNA CTG, with a mean of 6.8 foci per cell (**Figure 4C**). Most of the tested ONs significantly decreased the number of foci per cell, but with different efficiencies. siHTT, CAG/CUG, G2, A2F, and A2M reduced foci number to a mean of 7.9, 7.4, 7.3, 7.5, and 8.1 foci per cell, respectively. We did not observe significant foci alterations using ASO HTT. Next, we analyzed nuclear signals from the CTG probe (**Figure 4D**). All *HTT*-specific and CAG-specific ONs, except for LNA CTG, decreased level of detected RNA in the nucleus. siHTT and CAG/CUG reagents also triggered visible reductions in overall signal intensity from the cell. Differences between results obtained for measurement of nuclear level of transcript with RNA FISH and qRT-PCR after fractionation (Supplementary Figure S3A), are probably related to different specificity of the methods. Next, we compared results from foci analysis with total RNA level results obtained with qRT-PCR (**Figure 3A**). There was no clear correlation between the reduction in RNA levels and the RNA foci phenotype (Spearman correlation, *p* = 0.8603).

### Effects of ON-Based Reagents on Huntingtin Aggregates

**FIGURE 5:**
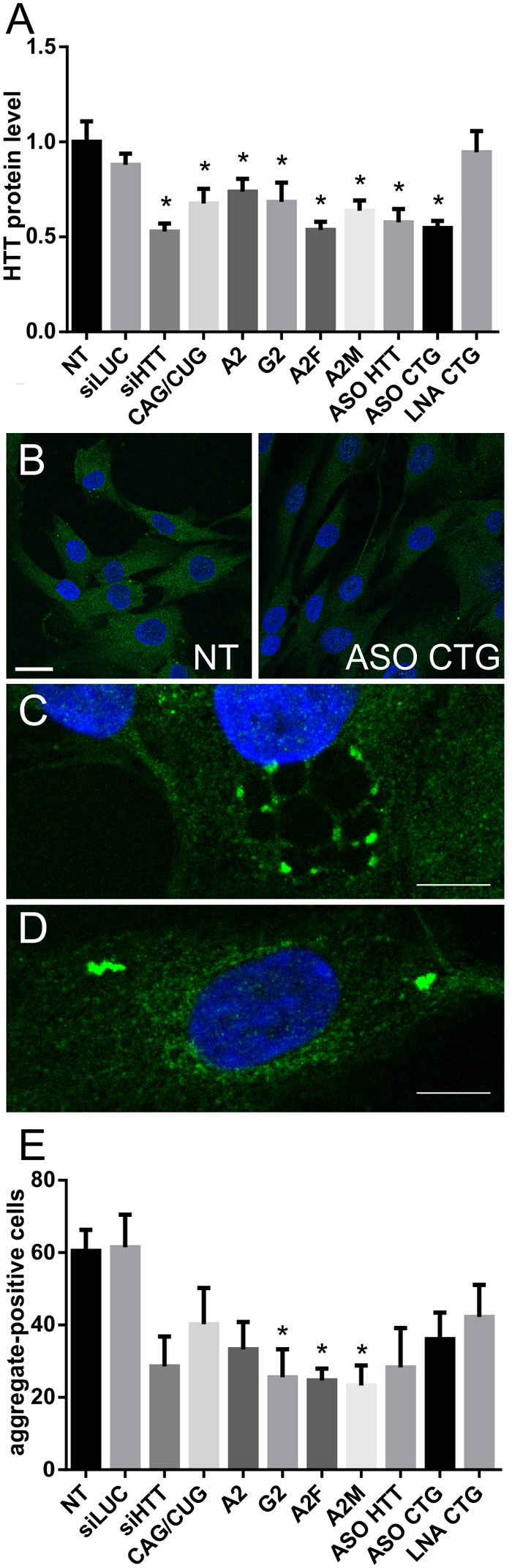
**Huntingtin level and presence of aggregates measured by IF after treatment of HD cells with ONs.** **(A)** Level of huntingtin based on IF performed in HD fibroblasts 48 h after transfection with 50 nM of the indicated ONs. **(B)** Representative images showing non-treated and ASO CTG-treated HD fibroblasts imaged with IF. Bar = 25 μm. **(C)** Representative image showing the localization of huntingtin around vesicles after transfection with ASO CTG. **(D)** Representative image showing the localization of huntingtin around the nucleus after transfection with ASO CTG. **(E)** Percent of aggregate-positive cells calculated for the same experiment as described in **(A)**. Statistical significance of changes after ON transfection was assessed in reference to measurements obtained for NT (non-treated) cells. DAPI staining (blue), HTT protein (green). Bar = 10 μm. The *p*-value is indicated with an asterisk (^∗^*p* < 0.05); graphs are presented with SEM values.

To further analyze the therapeutic potential of selected ONs, we performed IF experiments to detect huntingtin protein (both normal and mutant) in cells. The results of the microscopic analysis of HTT protein levels (**Figures 5A,B** and Supplementary Figure S5) were in agreement with results obtained by western blotting (**Figure 3B**). However, we could additionally observe differences in protein localization after ON treatment and the presence and number of mutant protein aggregates. Protein aggregates were observed in cells with or without RNA foci showing that there is no connection between formation of RNA foci and protein aggregates (Supplementary Figure S4). We observed that after treatment with ASO CTG, the protein tended to localize around the cytoplasmic vesicles containing ONs (**Figure 5C**). Moreover, in a substantial portion of cells, the protein localized around the nucleus after ON treatment (**Figure 5D**). Next, we measured the percentage of aggregate-positive cells (**Figure 5E**). With the decrease in mutant protein levels observed by western blotting, we also observed decreases in the number of protein aggregates. In untreated and siLUC-treated fibroblasts, we observed 1–3 aggregates in about 60% of cells. Protein aggregates localized mainly in the cytoplasm. Rarely, cells with a high number of cytoplasmic protein aggregates were observed and these cells also had nuclear aggregates (**Figure 2C**). We observed significant decrease in aggregate-positive cells up to 35–38% after treatment with A2M, G2, and A2F (**Figure 5E**).

## Discussion

Molecular processes involved in the manifestation and progression of polyQ diseases are not fully known; however, in addition to the roles of mutant proteins, the roles of mutant RNA in the pathomechanism of these diseases are increasingly recognized. RNA toxicity mechanisms include the formation of nuclear foci, protein sequestration within these foci triggering alternative splicing events and gene expression defects as well as aberrant biogenesis of small CAG-repeated RNAs (Galka-Marciniak et al., 2012; Fiszer and Krzyzosiak, 2013; Martí, 2016). Accordingly, the evaluation of new therapeutic approaches needs to be performed at both RNA and protein levels. In this paper, we proposed the application of microscopic techniques to monitor changes in the RNA foci phenotype as well as changes in the level and distribution of mutant proteins.

**FIGURE 6:**
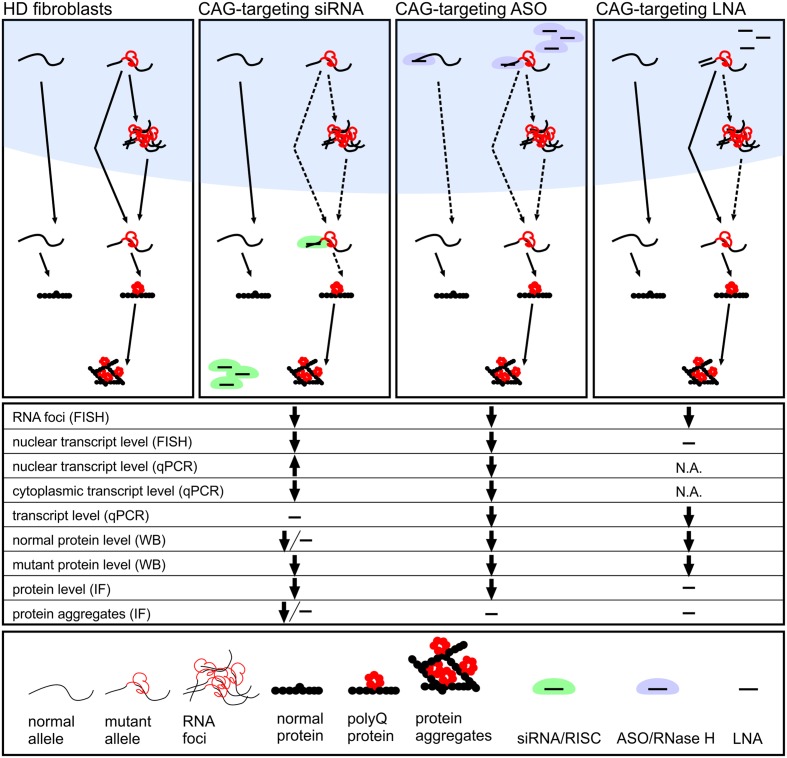
**Proposed activity mechanisms of investigated CAG-repeat targeting ON reagents: miRNA-like siRNAs, ASO and LNA designed to interact with CAG repeat tract.** We summarize results from the analyses of reagents localization (**Figure 1**), *HTT* gene expression level (**Figures 3**, **5** and Supplementary Figure S3), number of RNA foci per nucleus (**Figure 4**) and presence of huntingtin aggregates (**Figure 5**). miRNA-like siRNA (e.g., A2) act allele-selectively, decreasing preferentially the translation of mutant protein and reducing number of RNA foci. ASO interacts in the nucleus with both normal and mutant *HTT* mRNA lowering cellular level of RNA and protein. LNA exerts its activity in the nucleus decreasing slightly level of mRNA and reducing RNA foci number with only mild effect on translation.

As mutant RNA foci are found in the nucleus, it was not clear whether the ONs used would be able to affect RNA foci in any way. According to recent studies, RNA foci are not static, but rather dynamic structures, as shown for CGG and CUG repeats (Querido et al., 2011; Strack et al., 2013), and mutant transcripts were accessible to ON reagents. However, CAG RNA foci differ in morphology and localization from RNA foci found in other repeat expansion diseases (Wojciechowska and Krzyzosiak, 2011; Urbanek et al., 2016). Therefore, despite the successful deconstruction of RNA foci in other repeat expansion diseases (Supplementary Table S1), the ability to decrease CAG RNA foci phenotype was not obvious. Our results demonstrated that mutant *HTT* mRNA can be targeted by ON-based reagents in the nucleus, as the decrease in number of RNA foci was observed. This may be explained by the dynamic nature of foci, but may also indicate that the ONs examined in this study were incorporated into nuclear speckles or, alternatively, targeted mutant transcripts preventing their detention in nuclear speckles. The factors that distinguish the reagents and determine their potential to reduce RNA foci may be their cellular localization and mechanism of their action (**Figure 6** and Supplementary Table S3). Depending on the chemical composition of CAG repeat-targeting ONs and proteins that facilitate their binding to targets, they may possess different ability to bind transcripts localized in the nucleus and cytoplasm. Self-duplexing CAG-targeting siRNAs (A2) had little effect on RNA levels, but significantly decreased mutant protein levels and the number of RNA foci in analyzed time point. This effect was not explained by the spectrum of mechanisms specified in the section “Introduction.” This suggests that these siRNAs bind to mutant transcripts in the cell nucleus, preventing them from RNA foci formation as well as acting as translation repressor in the cytoplasm. The observed reductions in RNA foci may result also from earlier decreases in RNA levels which we cannot observe at the analyzed time point after reagent transfection, but was reported in our previous study (Fiszer et al., 2013). CAG-targeting LNA decreased RNA levels, but very mildly, and decreased RNA foci showing only slight influence on protein levels. These results are in agreement with the postulated LNA-modified ON function as an RNA blocker. LNA CTG interacting with CAG repeats was shown to be active also in neuronal cells and *in vivo*. LNA CTG treatment led to rescue of lowered levels of striatal markers and improved motor functions in HD mouse model. It was also shown that used ON was able to decrease number of foci-positive cells (Rué et al., 2016). LNA CTG may prevent mRNAs from RNA foci formation or release transcripts from foci by interfering with interactions between CAG tracts and proteins. The siRNA CAG/CUG, siHTT, and ASO CTG decreased both RNA and protein levels and also decreased RNA foci phenotype. This suggests that these ONs act, at least partially, in the nucleus. However, none of these ONs had an allele-selective effect on protein levels; therefore, they are not ideal for polyQ diseases treatment.

The comprehensive approach used in this study to compare various ONs in a single experimental model and conditions enabled us to draw general conclusions about the decrease in RNA foci phenotype. First, we observed that decreases in RNA foci number are not directly correlated with decreases in *HTT* mRNA levels. Using siHTT, we showed that despite a strong decrease in transcript levels, the influence on RNA foci was less than the effect observed for other tested ONs. These findings are in agreement with earlier reports indicating that siRNA against *C9orf72* RNA in ALS does not significantly alter RNA foci, despite a significant decrease of *C9orf72* RNA levels (Lagier-Tourenne et al., 2013). On the other hand, ASO CTG, which targeted the CAG repeat sequence, lowered the mRNA level, but also most effectively decreased the number of foci in cells. Similarly, ASOs targeting various regions of mutant *C9orf72* RNA, both repeats and specific sequence, led to decreases in the number of cells containing foci as well as the number of foci per single cell (Lagier-Tourenne et al., 2013). However, in our study, ASO targeting specific sequence did not substantially affect the RNA foci phenotype. We observed that CAG-targeting reagents were more effective in decreasing the number of RNA foci compared with other reagents. Repeat-targeting ONs are highly beneficial because they can be used for the treatment of other polyQ diseases, including several spinocerebellar ataxias (SCA1, SCA2, SCA3, and SCA7). Moreover, with suitable base substitutions and chemical modifications, these reagents can exhibit high allele-selectivity.

## Conclusion

Our results do not answer the question whether the observed reduction in foci number results mainly from the inhibition of new foci formation by ON binding to expanded CAG repeats in nucleoplasm or from deconstruction of already existing foci formed by mutant transcript which is detained in nuclear speckles. The presented results demonstrate that the ONs bind to mutant transcript already in cell nucleus and we hypothesize that this binding altering accessibility of CAG repeats in mutant transcript prevents its interaction with unidentified yet factor responsible for transcript nuclear detention.

## Author Contributions

WK, MU, and AF designed the study. MU performed FISH/IF and all microscopic analyses using ImageJ and prepared the figures and tables. AF performed western blotting, fractionation and real-time PCR experiments, including data analysis. MU and WK wrote the paper.

## Conflict of Interest Statement

The authors declare that the research was conducted in the absence of any commercial or financial relationships that could be construed as a potential conflict of interest.

## Abbreviations

ASO: antisense oligonucleotide
FISH: fluorescence *in situ* hybridization
HD: Huntington’s disease
IF: immunofluorescence
LNA: locked nucleic acid
ON: oligonucleotide
polyQ: polyglutamine
siRNA: small interfering RNA
