# Supplementary material for: Reduction of Huntington’s Disease RNA Foci by CAG Repeat-Targeting Reagents
Source: Front Cell Neurosci. 2017 Mar 28;11:82. doi: 10.3389/fncel.2017.00082 (PMC5368221; doi:10.3389/fncel.2017.00082)
Supplement: Supplementary file 1 [file Data_Sheet_1.pdf]

Supplementary Material

Reduction of Huntington's disease RNA foci by CAG repeat-targeting reagents

Martyna O. Urbanek [1], Agnieszka Fiszer [1], Wlodzimierz J Krzyzosiak [1]*

[1] Department of Molecular Biomedicine, Institute of Bioorganic Chemistry, Polish Academy of Sciences, Poznan, Poland

*** Correspondence:**

Wlodzimierz J Krzyzosiak

wlodkrzy@ibch.poznan.pl

1 Supplementary Figures and Tables

1.1 Supplementary Figures

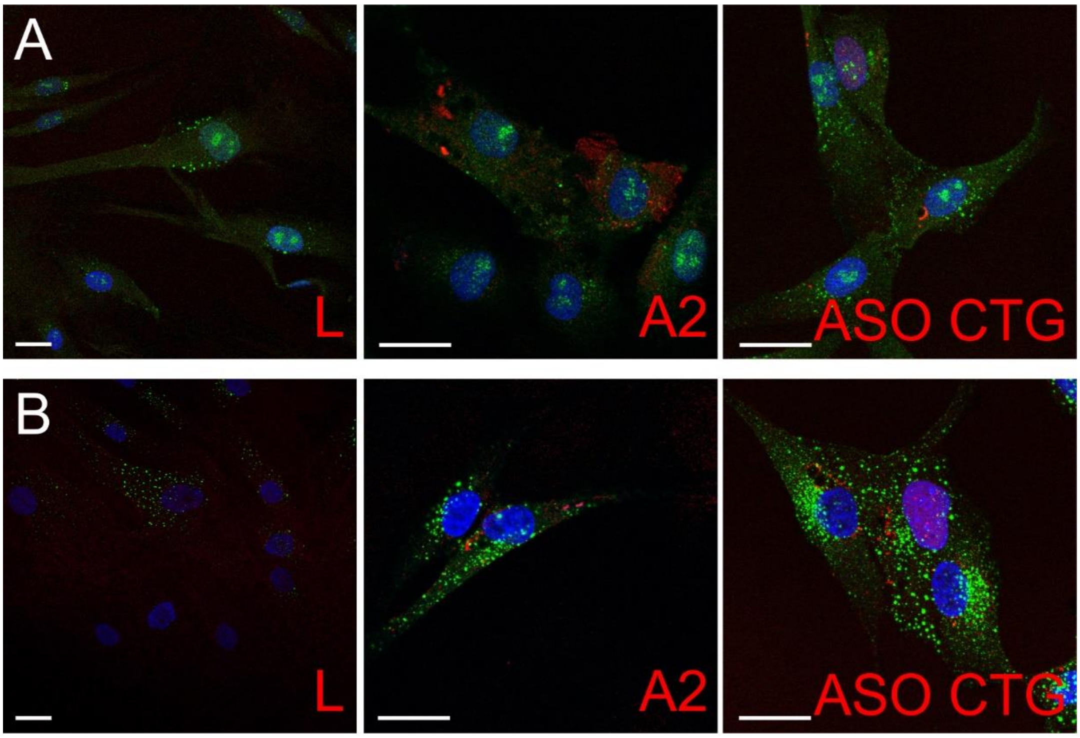

Supplementary Figure S1. ON-based reagents do not localize within endosomes. (A) ASO CTG and A2 ONs were imaged in HD fibroblasts with endosomes marked with anti-RAB5 antibody. **(B)** ASO CTG and A2 ONs were imaged in HD fibroblasts with endosomes marked with anti-EEA1 antibody. DAPI staining (blue), endosomal marker (green), ONs (red). L - cells treated with Lipofectamine only. Bar = 25 µm.

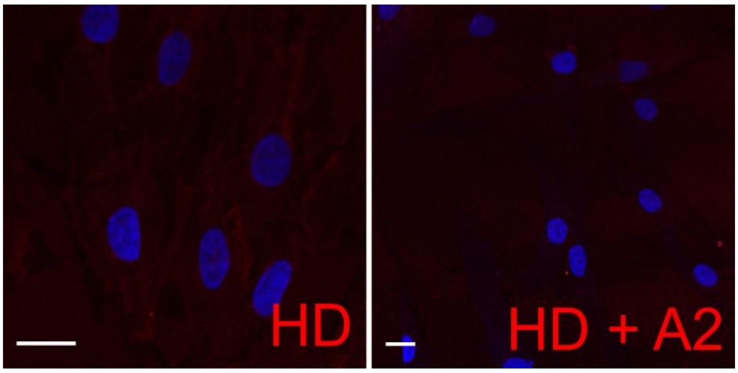

Supplementary Figure S2. RNase treatment lead to disappearance of RNA foci in HD fibroblasts. HD fibroblasts with control treatment and A2 were treated with RNase prior to RNA FISH. DAPI staining (blue), CAG repeats (red). Bar = 25 µm.

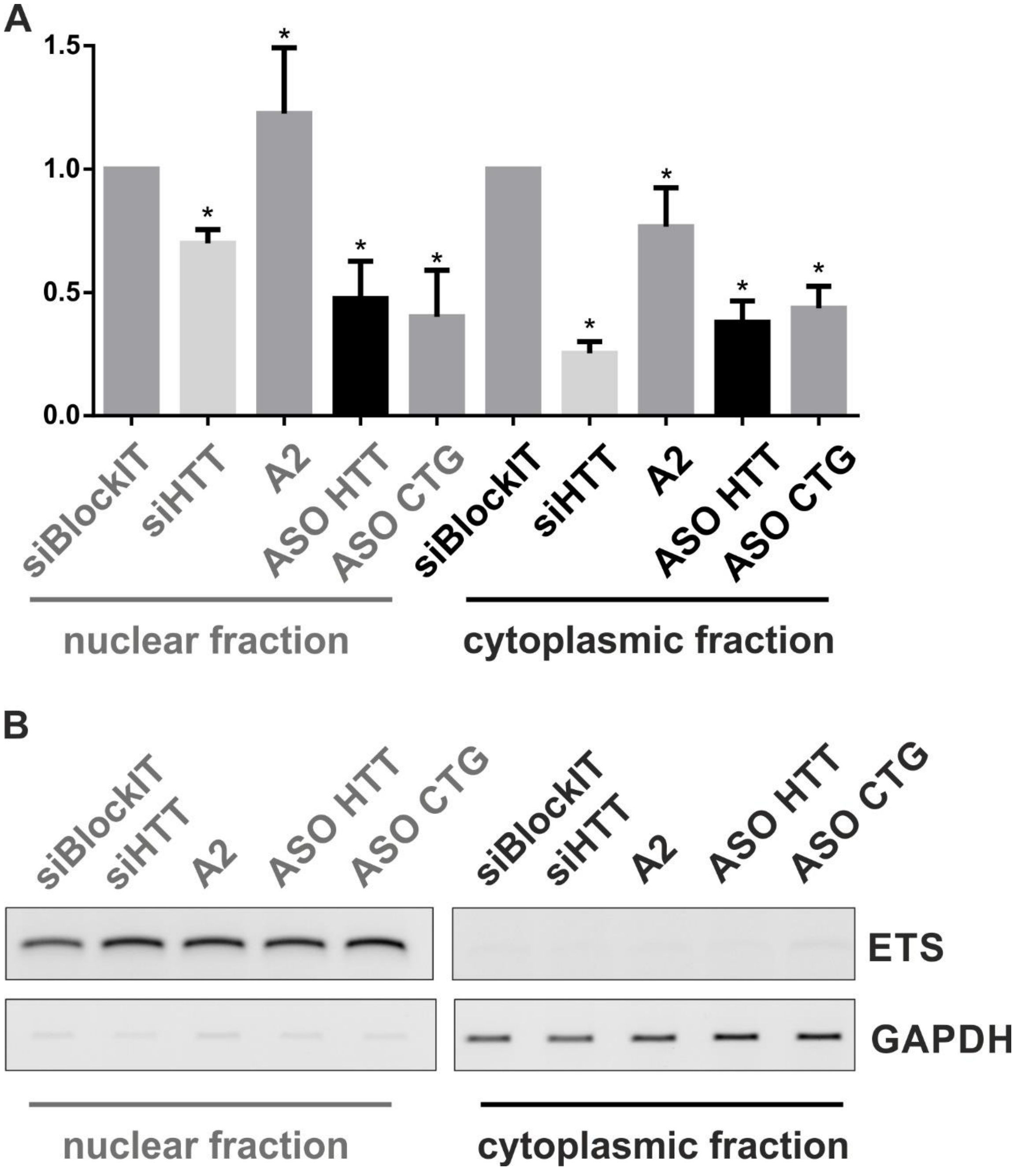

Supplementary Figure S3. Regulation of *HTT* mRNA level by ONs in nucleus and cytoplasm. (A) qRT-PCR analysis of fractionated *HTT* mRNA levels in HD fibroblasts after transfection with 50 nM of the indicated ONs. *HTT* cytoplasmic and nuclear level was normalized to the *GAPDH* and *U6* mRNA level, respectively. **(B)** Representative image of RT-PCR analysis of *GAPDH* and *ETS* expression levels in isolated fractions for the same experiment as in (A). In all samples expression level was referred to *HTT* expression in cells transfected with siBlockIT (set as 1). The p-value is indicated with an asterisk (* $p<0.05$); graphs are presented with standard deviation values.

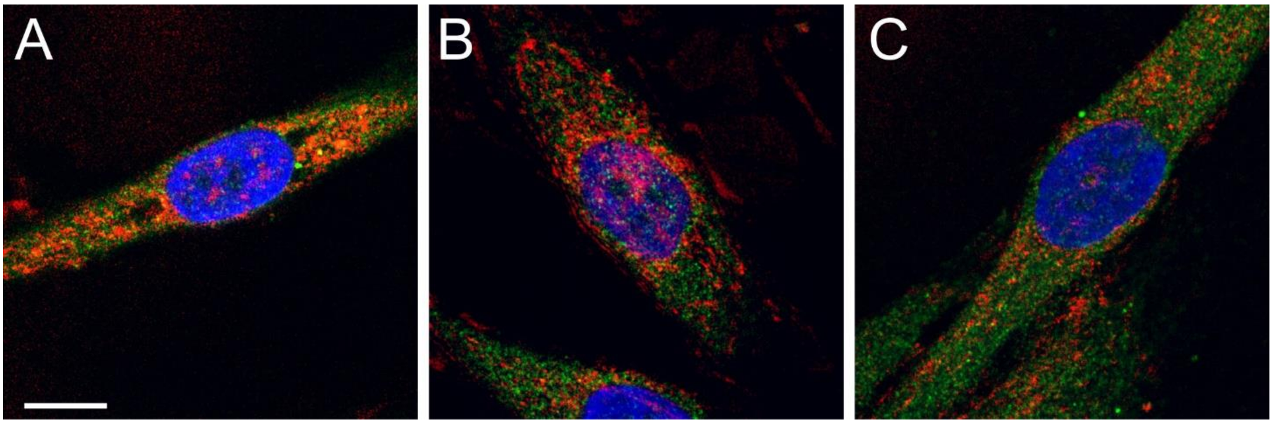

Supplementary Figure S4. RNA foci and protein aggregates presence in HD fibroblasts. Representative images of non-treated cells: **(A)** cell with CAG foci and HTT aggregates, **(B)** cell with CAG foci without protein aggregates, **(C)** cell without CAG foci with protein aggregates. DAPI staining (blue), HTT protein (green), CAG repeats (red). Bar = 10 µm.

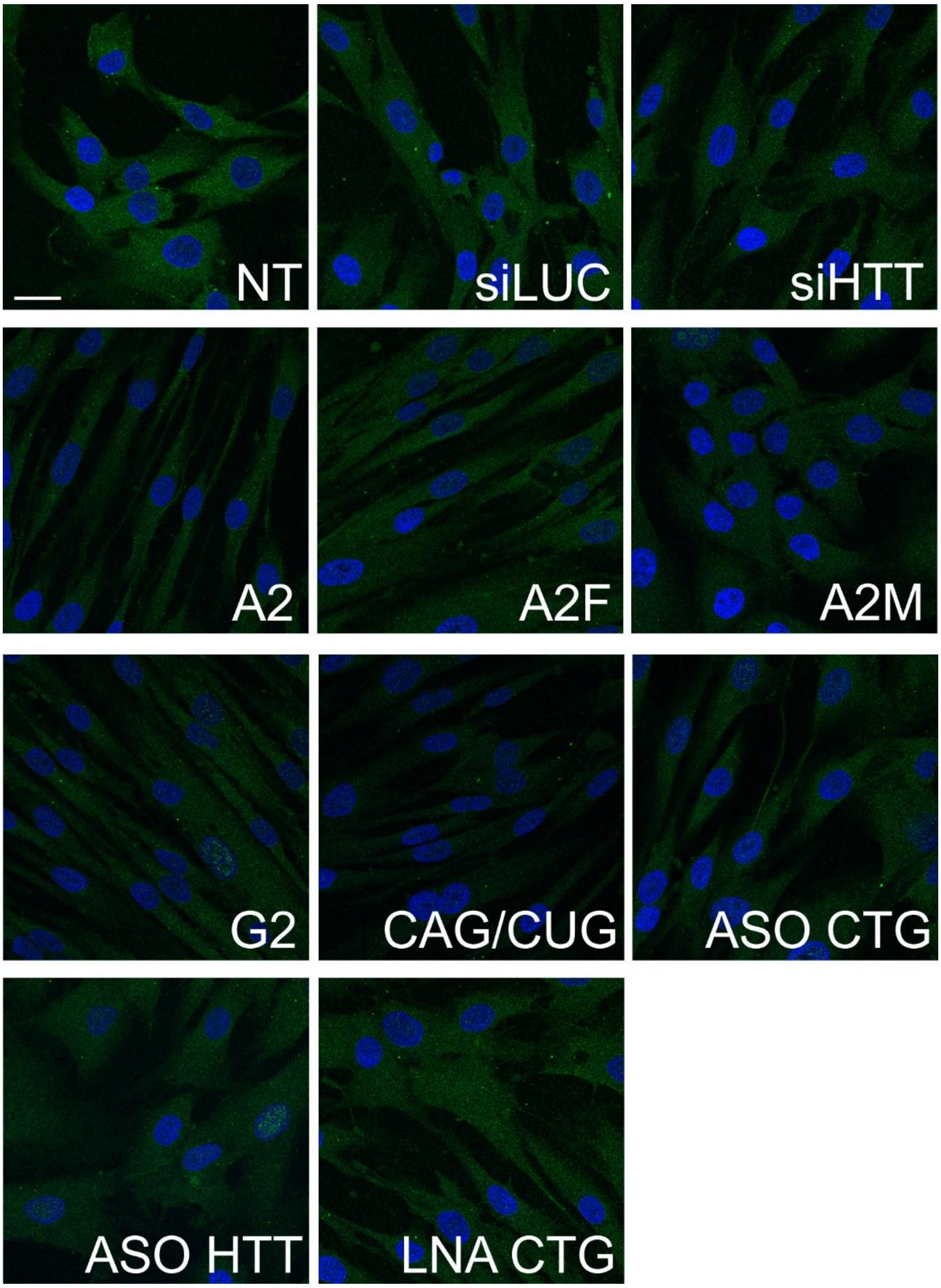

Supplementary Figure S5. Immunofluorescence of HTT protein. Representative images of HD fibroblasts treated with indicated ONs. DAPI staining (blue), HTT protein (green). Bar = 25 μm.

1.2 Supplementary Tables

Supplementary Table S1. Therapeutic approaches for RNA foci disintegration in various repeat-associated diseases.

**Disease (repeats)**	**Reagent**	**Effect**	**Reference**
**Small molecules**			
**DM1 (CUG)**	pentamidine	pentamidine reduced the number of RNA foci and decreased MBNL1 sequestration	(Warf et al., 2009)
**DM1 (CUG)**	spermine, 2, 5, 11, 9	ligands 2, 5, 11 and spermine did not significantly decrease foci number; ligand 9 triggered partial foci dispersion at lower concentrations and full foci dispersion at 50 μM	(Jahromi et al., 2013a)
**DM1 (CUG)**	ligand 1	ligand 1 disrupted RNA foci	(Jahromi et al., 2013b)
**DM1 (CUG)**	ligands C16 and C51	ligands C16 and C51 disrupted RNA foci	(Wojciechowska et al., 2014)
**DM1 (CUG)**	small molecules, e.g. hypericin, Ro 31-8220, gemcitabine, chromomycin A3	multiple small molecules disrupted RNA foci	(Ketley et al., 2014)
**DM1 (CUG)**	dilomofungin, lomofungin	dilomofungin increased the number and intensity of RNA foci; lomofungin did not affect RNA foci but increased levels of nucleoplasmic MBNL1	(Hoskins et al., 2014)
**DM1 (CUG)**	ligand 3	ligand 3 reduced RNA foci	(Wong et al., 2014)
**DM2 (CCUG)**	small molecules, e.g. hypericin, Ro 31-8220, gemcitabine, chromomycin A3	multiple small molecules disrupted RNA foci	(Ketley et al., 2014)
**DM2 (CCUG)**	small molecules, e.g., K-Ak and N3-K	multiple small molecules decreased the number of RNA foci	(Rzuczek et al., 2014)
**DM2 (CCUG)**	ligands 6, 11	all bisamidinium ligands that inhibited the MBNL1-RNA complex *in vitro* also disrupted the RNA foci	(Nguyen et al., 2014)
**FXTAS (CGG)**	ligand 1a	ligand 1a decreased the size and number of RNA foci	(Disney et al., 2012)
**FXTAS (CGG)**	ligand 1a	1a reduced the percentage of foci-positive cells	(Su et al., 2014)

**ALS (GGGGCC)**	ligands 1a, 2, 3	ligands 1a and 2, but not 3, significantly reduced the percentage of foci-positive cells; no change in the percentage of cells bearing antisense RNA foci was detected following 1a treatment	(Su et al., 2014)
**Oligonucleotide-based reagents**			
**DM1 (CUG)**	morpholino CAG25	CAG25 disrupted RNA foci	(Wheeler et al., 2009)
**DM1 (CUG)**	2′-*O*-methyl oligonucleotides	2′-O-methyl oligonucleotides reduced percentage of foci-positive cells	(Mulders et al., 2009)
**DM1 (CUG)**	hU7-$(CAG)_{15}$	hU7-$(CAG)_{15}$ disrupted RNA foci in a dose-dependent manner	(François et al., 2011)
**DM1 (CUG)**	ASO	CAG ASO disrupted RNA foci	(Larsen et al., 2011)
**DM1 (CUG)**	ASO	ASO disrupted RNA foci	(Wheeler et al., 2012)
**DM1 (CUG)**	CAG gapmers	LNA-CAG14 and LNA-CAG16 disrupted RNA foci	(Lee et al., 2012)
**DM1 (CUG)**	siCAG	siCAG completely disrupted RNA foci	(Sobczak et al., 2013)
**DM1 (CUG)**	LNA	LNA reduced the average number of RNA foci per cell	(Wojtkowiak-Szlachcic et al., 2015)
**ALS (GGGGCC)**	ASO	both ASO816 (which knocks down overall *C9ORF72* levels) and ASO061 (which specifically targets repeat-containing transcripts) disrupted RNA foci	(Sareen et al., 2013)
**ALS (GGGGCC)**	ASO	ASOs significantly decreased the number of foci-positive cells	(Lagier-Tourenne et al., 2013)
**ALS (GGGGCC)**	siRNA	siRNAs failed to disrupt RNA foci	(Lagier-Tourenne et al., 2013)
**HD (CAG)**	LNA	LNA-CTG significantly decreased the number of foci-positive cells	(Rué et al., 2016)
**Ribozyme**			
**DM1 (CUG)**	ribozyme	ribozyme disrupted RNA foci	(Langlois et al., 2003)

Supplementary Table S2. PCR primers.

Name	Sequence 5′-3′
GAPDH F	GAAGGTGAAGGTCGGAGTC
GAPDH R	GAAGATGGTGATGGGATTTC
HTT F	CGACAGCGAGTCAGTGAATG
HTT R	ACCACTCTGGCTTCACAAGG
U6 F	GTGCTCGCTTCGGCAGCACA
U6 R	GGAACGCTTCACGAATTTGCGTGTCAT
ETS F	GTCCCCTCGTCTCTCCTCTC
ETS R	ACAGCGAGGGCTGTCTGC

Supplementary Table S3. Cellular effects of tested ONs. N – nucleus, C – cytoplasm, N.A. – not analyzed.

ON	Main mechanism of action	Main localization	RNA foci (FISH)	Nuclear mRNA level (FISH)	Nuclear mRNA level (qPCR)	Cytoplasmic mRNA level (qPCR)	mRNA level (qPCR)	Protein level (WB)<br><br>normal/ mutant	Protein level (IF)	Protein aggregates (IF)
**CAG repeat tract-specific**										
**A2**	RNAi / miRNA	C	⇩	⇩	⇧	⇩	-	⇩/⇩	⇩	-
**G2**	RNAi / miRNA	C	⇩	⇩	N.A.	N.A.	⇧	⇩/⇩	⇩	⇩
**A2F**	RNAi / miRNA	C	⇩	⇩	N.A.	N.A.	⇧	- /⇩	⇩	⇩
**A2M**	RNAi / miRNA	C	⇩	⇩	N.A.	N.A.	⇧	⇩/⇩	⇩	⇩
**CAG/CUG**	RNAi	C	⇩	⇩	N.A.	N.A.	⇩	⇩/⇩	⇩	-
**LNA CTG**	blocker	N.A.	⇩	-	N.A.	N.A.	⇩	⇩/⇩	-	-
**ASO CTG**	RNaseH	N/C	⇩	⇩	⇩	⇩	⇩	⇩/⇩	⇩	-
***HTT* sequence-specific**										
**siHTT**	RNAi	N.A.	⇩	⇩	⇩	⇩	⇩	⇩/⇩	⇩	-
**ASO HTT**	RNaseH	N.A.	-	⇩	⇩	⇩	⇩	⇩/⇩	⇩	-
